# Diagnostic Accuracy of Urine Dipsticks for Urinary Tract Infection Diagnosis during Pregnancy: A Retrospective Cohort Study

**DOI:** 10.3390/antibiotics13060567

**Published:** 2024-06-19

**Authors:** Dominique E. Werter, Caroline Schneeberger, Suzanne E. Geerlings, Christianne J. M. de Groot, Eva Pajkrt, Brenda M. Kazemier

**Affiliations:** 1 Department of Obstetrics and Gynaecology, Amsterdam UMC Location University of Amsterdam, 1105 AZ Amsterdam, The Netherlands; 2 Amsterdam Reproduction and Development Research Institute, 1105 AZ Amsterdam, The Netherlands; 3 Department of Medical Microbiology and Infection Control, Amsterdam UMC Location University of Amsterdam, 1105 AZ Amsterdam, The Netherlands; 4 Nivel (Netherlands Institute for Health Services Research), 3513 CR Utrecht, The Netherlands; 5 Department of Internal Medicine, Division of Infectious Diseases, Amsterdam Institute for Infection and Immunology, 1105 AZ Amsterdam, The Netherlands; 6 Amsterdam Public Health Research Institute, Amsterdam UMC Location University of Amsterdam, 1105 AZ Amsterdam, The Netherlands; 7 Department of Human Genetics, Amsterdam UMC Location Vrije Universiteit Amsterdam, 1081 HV Amsterdam, The Netherlands; 8 Department of Obstetrics and Gynaecology, Wilhelmina Kinderziekenhuis, University of Utrecht, 3584 CX Utrecht, The Netherlands

**Keywords:** urinary tract infections, pregnancy, diagnostic accuracy, urine dipstick

## Abstract

Objective: Urinary tract infections (UTIs) represent the most prevalent infections among pregnant women. Many pregnant women experience frequent voiding or lower abdominal pain during pregnancy due to physiologic changes. Due to the possible consequences of a UTI in pregnancy, pregnant women are more often tested for UTIs. This study aimed to assess the diagnostic accuracy of dipsticks in diagnosing UTIs in pregnant women while using the urine culture as the reference standard. Study design: This was a retrospective cohort study, conducted at two academic hospitals in the Netherlands among pregnant women. Pseudonymized data were collected from patient files. The results of the urine dipstick and the urine culture in pregnant women were linked. Additionally, nitrofurantoin prescriptions were linked to culture results. A positive urine culture was considered the reference test for a UTI. Results: Between 1 January 2017 and 28 February 2021, a total of 718 urine samples with leukocyte esterase dipstick results within 24 h of the urine culture were analyzed. Of these samples, a nitrite dipstick result was also available in 337 cases. Only 6.8% of the 718 urine samples yielded positive cultures. The sensitivity and specificity of leukocyte esterase were 75.5% and 40.4%, respectively; for nitrite, 72.0% sensitivity and 73.4% specificity were found. When at least one of the two tests was positive, the sensitivity and specificity were 92.0% and 27.9%, respectively. When both tests were positive, the sensitivity and specificity were 52.0% and 82.7%, respectively. In only 16.8% of the women to whom nitrofurantoin was prescribed, the urine cultures returned positive using a cut-off of 10^5^ colony forming units/mL. Conclusion: The diagnostic performance of leukocyte esterase, nitrite, or their combination in clinical practice is lower than previously reported in study settings among pregnant women. A significant proportion of women treated with nitrofurantoin were found to have no UTI, suggesting potential over-prescription based on dipstick test results. Healthcare providers should be aware of this reduced performance in clinical practice and carefully weigh the risks of antibiotic treatment by suspicion of a UTI against the possibility of delayed treatment awaiting culture results in individual patients.

## 1. Introduction

Urinary tract infections (UTIs) are the most common infections during pregnancy. Worldwide, the prevalence of UTIs in pregnancy range from approximately 2.3 to 15% [1,2,3]. UTIs are associated with preterm birth [4,5], low birth weight and pre-eclampsia; therefore, treatment is started at a low threshold [5,6].

The indicated treatment for UTIs during pregnancy is antibiotics. However, the use of antibiotics can have negative consequences for both the pregnant woman and the unborn child. On the maternal side, negative consequences of antibiotic use include side effects, allergic reactions, Stevens–Johnson syndrome and kidney failure [7,8]. The possible negative impact of antibiotic use in pregnancy for the fetus involve birth defects (associated with nitrofurantoin), necrotizing enterocolitis in the neonate (associated with amoxicillin–clavulanic acid) or cerebral palsy (associated with erythromycin or amoxicillin–clavulanic acid) [9,10,11]. Another possible consequence is that by altering the maternal microbiome with antibiotic use, the neonatal microbiome can be changed. This could be a trigger in the development of conditions like asthma, diabetes, obesity, cardiovascular diseases, neurological disorders and inflammatory bowel diseases [12,13,14].

Exposure to antibiotics is also one of the drivers for antimicrobial resistance [15].

Typical UTI symptoms are less well defined in pregnancy. Physiological changes during pregnancy can mimic UTI symptoms, such as frequent voiding due to the fetal pressure on the maternal bladder [16,17]. Previous research reported that 12% of pregnant women reported dysuria and 20% frequent voiding without the presence of bacteriuria [18]. Symptoms can also be non-specific, such as, for example, uterine contractions which can be a symptom of a UTI but also occur in physiologic pregnancies [16,19]. The combination of more frequent symptoms without a UTI and the presence of non-specific symptoms leads to a lower a priori chance of actually having a UTI in pregnancy (1–11%) compared to non-pregnant women (49–61%). Therefore, there is probably a different diagnostic accuracy of diagnostic tests for UTIs in the pregnant population [20,21,22]. Currently, there is no available evidence to determine which symptoms are (the most) predictive for UTIs in pregnancy [23].

For a long time, only the presence of a significant amount of bacteria in urine (bacteriuria) was considered a UTI. Now, it is understood that bacteriuria can exist without causing an infection, similar to bacteria on the skin or in the gut (colonization). In pregnancy, asymptomatic bacteriuria can be present too. Asymptomatic bacteriuria has a smaller negative impact compared to a UTI in pregnancy, and therefore, it is arguable if pregnant women with asymptomatic bacteriuria should be treated [24].

The first screening test for a UTI is often a urine dipstick, as the results can be read within minutes. Urine dipsticks demonstrate if nitrite (converted from nitrate by the enzyme nitrate reductase produced by a selection of Gram-negative bacteria) and leukocyte esterase (an enzyme released by neutrophils and macrophages) are present in the urine [25,26]. However, in pregnancy, the presence of leukocytes in urine can be physiological [27]. The dipstick test is often followed by a urine culture, which can take days to yield results. Both the nitrite dipstick test and the urine culture indicate the presence of bacteria (bacteriuria). The leukocyte dipstick test, on the other hand, indicates the presence of infectious cells. Previous studies have mainly been conducted in women with asymptomatic bacteriuria, not in women with a UTI [28]. We expect a different sensitivity and specificity in women with a UTI.

In the Netherlands, antibiotic treatment is initiated when the nitrite dipstick test is positive [19]. General practitioners in the Netherlands initiate treatment directly when nitrite and/or leukocyte screening is positive [29]. In the Netherlands, the first-line treatment for uncomplicated UTIs in pregnancy is nitrofurantoin (unless there is an expected birth in the upcoming weeks). Nitrofurantoin is not prescribed for other indications in contrast to, for instance, amoxicillin–clavulanic acid. That is why the use of nitrofurantoin is specific to the treatment of UTIs [30].

In this study, we investigated the diagnostic accuracy of leukocyte esterase and nitrite dipstick test results for UTIs in daily practice in pregnant women. The urine culture results will be used as a reference test. In addition, we will evaluate the diagnostic process itself and analyze the number of positive cultures in pregnant women who received nitrofurantoin for a suspected UTI.

## 2. Results

Between 1 January 2017 and 28 February 2021, a total of 718 urine samples with one or more dipstick results within 24 h of the culture from pregnant women were available for analysis.

### 2.1. Dipstick

#### 2.1.1. Leukocyte Esterase

In total, 718 urine samples had leukocyte esterase dipstick results available. Of these samples, 60.7% were considered positive for leukocyte esterase (Table 1). Only 6.8% of all urine samples contained 1 or 2 bacteria ≥ 10^5^ CFU/mL, and were therefore considered positive.

The sensitivity of leukocyte esterase was 75.5% and the specificity was 40.4% for pregnant women tested for a suspected UTI.

With a cut-off value of ≥10^4^ CFU/mL, the sensitivity and specificity were comparable (Supplementary Table S1). The pathogens identified were primarily *Escherichia coli* (71%) and *Klebsiella pneumoniae* (20%) (Supplementary Table S2). When the cut-off value of ≥10^4^ CFU/mL was used, there was a higher variety of bacteria identified (Supplementary Table S2).

#### 2.1.2. Nitrite

In total, 337 urine samples had nitrite dipstick results. Of these urine samples, 28.4% were nitrite-positive (Table 2). When a cut-off of ≥10^5^ CFU/mL was applied to the samples, the sensitivity of nitrite was 72.0% and the specificity was 73.4%.

The sensitivity was lower when a cut-off value of ≥10^4^ CFU/mL was used, but the specificity was similar (Supplementary Table S3).

#### 2.1.3. Leukocyte Esterase and/or Nitrite

In total, 337 urine samples had an available result of both leukocyte esterase and nitrite. When a cut-off of ≥10^5^ CFU/mL was used, the sensitivity of a positive leukocyte esterase and/or nitrite was 92.0% and the specificity was 27.9% (Table 3). If both the nitrite and the leukocyte esterase were positive, the sensitivity was 52.0% and the specificity 82.7% (Table 4).

With a cut-off value of ≥10^4^ CFU/mL, sensitivity and specificity were similar in the group in which one of the tests had to be positive. If both leucocyte esterase and nitrite had to be positive to consider the test as a positive screening result, the sensitivity decreased from 52.0% to 35.1%; the specificity did not alter much (Supplementary Tables S4 and S5).

### 2.2. Nitrofurantoin

**Figure 1:**
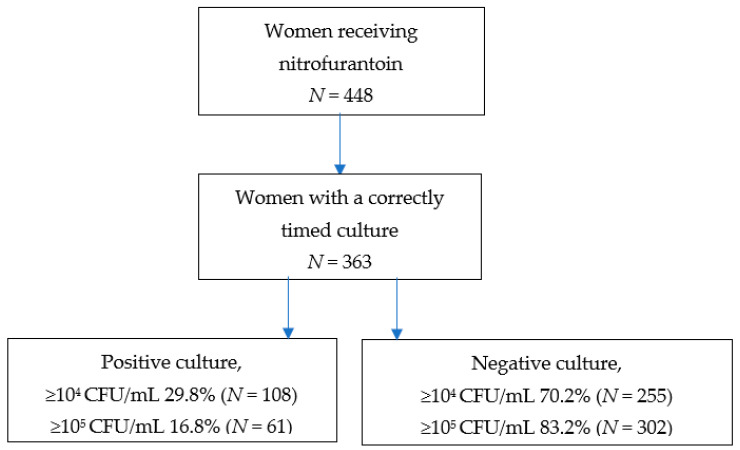
Women receiving nitrofurantoin and their cultures.

In our cohort, 448 women received nitrofurantoin during pregnancy, with 417 receiving it only once. Of these, 336 women had a culture performed 7 days before and up until 2 days after the moment nitrofurantoin was prescribed (Figure 1). In 95% (320 women), the urine sample was sent to the laboratory at the same date as the nitrofurantoin was prescribed.

In case of a cut-off of ≥10^5^ CFU/mL, in 16.8% of the correctly timed urine samples, the culture was positive in women who received nitrofurantoin. With a cut-off of ≥10^4^ CFU/mL, the percentage of positive urine samples was 29.8% in women who received nitrofurantoin (Figure 1).

## 3. Methods

### 3.1. Study Design

We conducted a retrospective cohort study in the Netherlands. Data were extracted from electronic patient files of pregnant women presenting at the Amsterdam University Medical Centers located at the Academic Medical Centre and VU Medical Centre between January 2017 and February 2021.

### 3.2. Inclusion

Pregnant women were identified using the “Diagnosis Treatment Combination On the way to Transparency” (DOT) code, assigned to specific diagnoses related to pregnancy and birth. In this study sample, all diagnoses were related to pregnancy and birth. Women were included if they had any laboratory recording and a pregnancy-related DOT code. The collected data were pseudonymized and contained information on whether or not the patient was pregnant, urinary laboratory results and prescribed antibiotics. The data were extracted from either the patients admitted to the hospital or patients visiting the outpatient clinic. Unfortunately, due to privacy restrictions, the available data were very limited. The baseline characteristics, pregnancy characteristics or clinical characteristics such as symptoms were not available.

### 3.3. Databases

The laboratory results of the dipstick, the results of the urinary culture and the prescribed antibiotics were extracted, and two databases were created:

1. The results of the urine dipstick (nitrite and leukocyte esterase) and the results of the urine culture (reference test) were linked. The urine dipstick was linked to the urine culture if the dipstick was performed within 24 h of the culture in the same woman.
2. Nitrofurantoin use was linked to the available urine culture in a pregnant woman under the condition that the urine culture was performed from seven days before the prescription date until two days after the prescription date.

### 3.4. Definitions

#### 3.4.1. Urine Dipstick

Midstream urine samples were collected in a clean jar after perineal cleansing with water and cotton after instructions from a nurse. Urine samples were stored in a refrigerator at the outpatient clinic before being transported to the laboratory for a maximum of 24 h.

Nitrite and leukocyte esterase were measured using a urine dipstick. For nitrite, the result was either negative or positive. For leukocyte esterase, minus or a +/− was considered negative (corresponding to 0–15 leukocytes per µL). One or two pluses was considered positive (corresponding to 70 leukocytes or more per µL).

Depending on the requested test, the outcome of a urine dipstick was reported either with leukocyte esterase only or with both the leukocyte esterase and nitrite results.

#### 3.4.2. Urine Culture

Screening for asymptomatic bacteriuria is rare in the Netherlands. Screening for asymptomatic bacteriuria was not common practice at our hospital either. Therefore, in general, a urine culture is only performed when a pregnant woman has complaints of a UTI or after completion of antibiotics for a UTI [19]. We made the assumption that none of the urine cultures were performed for screening based on accepted clinical practice and local guidelines. Therefore, we considered a positive urine culture the reference test for a UTI. Following international guidelines, we considered a urine culture positive when there were 1 or 2 bacteria present for ≥10^5^ colony forming units (CFU)/mL [31]. A sub-analysis was performed with a bacterial count of ≥10^4^ CFU/mL since it is known that *Escherichia coli* at lower colony counts in midstream urine can be indicative of a UTI [32]. The standard operating procedure of the laboratory was followed. The urine culture was performed on the same day as the urine dipstick.

### 3.5. Analysis

The sensitivity, specificity, positive predictive value, negative predictive value, total accuracy, positive likelihood ratio and negative likelihood ratio were calculated for leukocyte esterase and nitrite dipstick results separately and combined. In case more than one dipstick was performed within 24 h, the results of the first dipstick were used. Sensitivity, specificity, disease prevalence, positive and negative predictive values are expressed as percentages. The confidence intervals for sensitivity, specificity and accuracy are “exact” Clopper–Pearson confidence intervals.

The confidence intervals for the likelihood ratios were calculated using the log method and confidence intervals for the predictive values are the standard logit confidence intervals given by Mercaldo et al. (2007) [33,34].

We conducted an additional analysis. We analyzed all women who used nitrofurantoin (nitrofurantoin is only used to treat UTIs in the Netherlands) in pregnancy and matched women if they had a culture performed 7 days before and up until 2 days after nitrofurantoin was prescribed. The number of women with a positive urine culture and considered legitimate reason for antibiotics is expressed as *N* with %.

All analyses were performed using IBM SPSS statistics 25.

### 3.6. Ethical Approval

The Medical Ethics Review Committee of the Academic Medical Centre reviewed the data collection and waived an official approval (reference number W21_475 # 21.527app).

## 4. Discussion

Of the 718 urine samples taken in this study, only 6.8% had a positive urine culture. Of the women with a positive leukocyte esterase result (*N* = 436), only 8.5% had a UTI. Of the women with a positive nitrite result (*N* = 101), only 17.8% had a positive culture. In case of either a positive leukocyte esterase and/or a nitrite (*N* = 248), only 0.9% had a positive culture. When both nitrite and leukocyte esterase were positive (*N* = 67), only 19.4% had a positive culture. In the women who received nitrofurantoin, only 16.8% had a positive urine culture. The diagnostic performance of the leukocyte esterase dipstick test, nitrite dipstick test or the combination of tests in clinical practice was lower than previously reported within study settings in pregnant women. This discrepancy in daily practice could lead to the unnecessary treatment of suspected UTIs if clinicians do not wait for the results of the urine culture, which is often the case.

### 4.1. Strengths and Limitations

A strength of this study is that our data were collected during everyday practice, enhancing the generalizability of our results. Additionally, compared to most other studies, the analysis was performed in a relatively large cohort of pregnant women; other studies included 100–200 pregnant women [18,35].

However, there are some limitations to this study. Firstly, there are no available data on demographics, gestational age and the signs and symptoms of the women enrolled in this study. However, in the Netherlands in general, and at these hospitals in particular, no screening takes place for asymptomatic bacteriuria, in line with the local protocol. This makes it more plausible that the urine tests were performed because a UTI was suspected. Studies in the female non-pregnant population show that the presence of symptoms related to UTIs is important to predict a UTI [36]. In pregnant women, these symptoms are both less distinct and more often already present as a result of the pregnancy itself (e.g., frequency). Unfortunately, in this study, no data on the clinical symptoms were available. However, with the current daily practice of not testing for asymptomatic bacteriuria, we cannot rule out that this never happened because we do not have the information of the signs and symptoms the pregnant woman presented with. This could lead to a lower a priori chance of UTIs, influencing the diagnostic accuracy of the tests. Since the variation in infection risk and symptomatology across pregnancy trimesters, conducting a sub-analysis of the data distributed by trimester, would be beneficial, unfortunately, these data were not available.

Additionally, we did not have extra information to confirm if the urine samples were adequate.

In practice, the diagnostic work-up for suspected UTIs was not the same for all pregnant women. For 718 urine samples, at least one leukocyte esterase dipstick result was available in comparison to only 337 urine samples for which a nitrite dipstick result was also available. The test was conducted in a uniform manner; nonetheless, not all findings are disclosed, depending on the requested test outcome. There could be a report bias.

Another limitation is that we only have data from two academic hospitals. In the Netherlands, not all women receive prenatal care at a hospital; the majority of low-risk pregnancies receive their antenatal care with a local midwife. In case of complaints of a UTI, they could be either referred to a hospital or a general practitioner. General practitioners in the Netherlands can also perform urine dipsticks and cultures if indicated. Therefore, the generalizability of our results is limited to hospitals. At hospitals, there could be an over-presentation of high-risk pregnancies, for example, with abnormalities from the urinary tract. This may influence the results of the performance of diagnostic tests. The guideline for general practitioners states nitrofurantoin as the first choice. The guideline for obstetrics states either nitrofurantoin or amoxicillin–clavulanic acid. In this study, we assumed that these treatments do not differ too much [19,29].

Finally, we selected the group of pregnant women that received nitrofurantoin. In the Netherlands, in general, we treat women with a (threatened) birth and after 36 weeks of gestational age with a UTI with amoxicillin–clavulanic acid and not with nitrofurantoin. With this decision, a selection bias was introduced. The guideline adherence for prescribing the correct antibiotics for a UTI in the general population is relatively good in the Netherlands (65–75%). Unfortunately, there is no separate date available for guideline adherence in pregnant women only [30,37]. When we extrapolate these findings to our study, it would mean that most women do receive nitrofurantoin for their UTI in pregnancy nor another antibiotic. In addition, in a national survey, 16 out of 19 hospitals implemented the suggested antibiotics by the national guidelines into their local guideline. As mentioned before, amoxicillin–clavulanic acid is prescribed for other indications which could not be deduced from our dataset. That is why we only used nitrofurantoin in our study.

### 4.2. Interpretation of Data

The current Dutch guideline for obstetrics advises to start the treatment of UTI in case of a positive nitrite. This is based on a study by Kodikara et al. [18]. In this study, there were only eight positive cultures in 205 pregnant women using a cut-off value of ≥10^5^ CFU/mL (3.9%). The nitrite dipstick detected six out of the eight positive cultures.

They found 1.0% (two positive nitrite dipsticks out of 205 cultures) false positives when using nitrite dipstick test results, whereas in our study, the number of women with a false positive nitrite was much higher (23.1%). The most important difference between the studies is the fact that Kodikara et al. tested women for asymptomatic bacteriuria, whereas in our study, we assumed that women had complaints of a UTI when they were tested. Remarkably, the number of women with a positive culture is quite low in our sample (6.8%), indicating that the majority of women tested for a UTI during pregnancy in practice did not have a UTI at all.

A systematic review looking into the diagnostic accuracy of dipsticks in asymptomatic bacteriuria found a pooled sensitivity for nitrite of 0.55 (95% CI 0.42–0.67) and a specificity of 0.99 (95% CI 0.98–0.99). However, the outcome of the different tests was quite heterogeneous [28]. The majority of the studies in this review included only asymptomatic women which is different to our population [35,38,39,40].

Not all uropathogens produce nitrite. Nitrate is converted to nitrite by the most common uropathogens like *Escherichia coli*; however, *Streptococcus agalactiae* does not produce nitrite and is a known cause of a UTI in pregnancy, therefor solely a negative nitrite is not enough to exclude a UTI. On the other hand, nitrite can also be produced by bacteria causing sexually transmittable infections like *Chlamydia trachomatis,* and is not specific to UTIs.

The relatively low specificity of leukocyte esterase dipstick results found in our study and previous studies could be explained because, in pregnancy, women can have physiological pyuria [27]. In addition, pregnant women have more vulvovaginal candidiasis compared to the general female population, which could result in leukocytes in urine when the vulva is not washed before taking the urine sample [41].

When treatment is started based on the results of the dipstick, and more specifically based on the leukocyte esterase, nitrite or nitrite and/or leukocyte esterase dipstick test results, more women are overtreated than correctly treated. This scenario is further supported by the finding that only 16.8% of women treated with nitrofurantoin had a positive urine culture. This may have serious consequences for both mother, child and society. Maternal risks regarding antibiotic use include a small risk of (anaphylactic) allergic reactions, Stevens–Johnson syndrome and kidney failure. In addition, antibiotics are known for their alterations of the microbiome [7,8,42,43,44,45]. There is an association between intra-uterine antibiotic exposure and adverse neonatal outcomes like cerebral palsy, malformations and early onset sepsis with antibiotic-resistant micro-organisms [8,43,44]. Finally, antibiotic use is one of the big contributors to antimicrobial resistance [15]. Therefore, the prescription of antibiotics should not be performed without a clear indication, especially not in pregnancy.

We assume that clinicians in general have a low threshold for testing for UTI and additionally prescribing antibiotics in pregnancy because of the previously found associations with preterm birth and the profound consequences of a preterm birth [4]. However, it is unknown whether a UTI directly causes a preterm birth or that women with a UTI are a group of women who are at an increased risk for a preterm birth due to common risk factors regardless of the moment of the UTI.

It is currently unknown if the risk of preterm birth or pyelonephritis is increased in pregnancy if treatment is withheld for a few days until the culture results are available. In non-pregnant women, placebo arms of randomized trials have shown that almost half of women presenting with UTI symptoms will spontaneously recover in one week without an increased risk of developing pyelonephritis [46,47,48]. However, it is uncertain if this is also applicable in pregnant women, since the immune system in pregnancy is altered [49,50]. This should be investigated in future studies.

## 5. Conclusions

Our study indicates that the diagnostic performance of leukocyte esterase and/or nitrite dipstick tests in clinical practice for pregnant women is lower than previously reported. The interpretation of the sensitivity and specificity found in this study for the dipstick can be utilized by individual healthcare providers to assess whether or not to initiate antibiotic treatment for their individual patients depending on the clinical presentation and comorbidities. Future research should explore if there are risks present by waiting for the results of the culture before initiating antibiotic treatment, and if these risks outweigh the high number of unnecessary antibiotic prescriptions in pregnancy.

## Figures and Tables

**Table 1:** Performance of the leukocyte esterase dipstick test in pregnant women (*N* = 718).

	Positive Leukocyte Esterase	Negative Leukocyte Esterase	Total
Positive culture	37	12	49
Negative culture	399	270	669
Total	436	282	718
		**Culture 10^5^ CFU/mL**	**95% CI**
Sensitivity		75.5%	61.1–86.7%
Specificity		40.4%	36.6–44.2%
Positive likelihood ratio		1.27	1.07–1.50
Negative likelihood ratio		0.61	0.37–1.00
Positive predictive value		8.5%	7.3–9.9%
Negative predictive value		95.7%	93.2–97.4%
Total accuracy		42.8%	39.1–46.5%

**Table 2:** Performance of the nitrite dipstick test in pregnant women (*N* = 337).

	Positive Nitrite	Negative Nitrite	Total
Positive culture	18	7	25
Negative culture	83	229	312
Total	101	236	337
		**Culture 10^5^ CFU/mL**	**95% CI**
Sensitivity		72.0%	50.6–87.9%
Specificity		73.4%	68.1–78.2%
Positive likelihood ratio		2.71	1.99–3.68
Negative likelihood ratio		0.38	0.20–0.72
Positive predictive value		17.8%	13.8–22.8%
Negative predictive value		97.0%	94.6–98.4%
Total accuracy		73.3%	68.3–78.0%

**Table 3:** Performance of the leukocyte esterase and/or nitrite dipstick test in pregnant women (*N* = 337).

	Positive Leukocyte Esterase and/or Nitrite	Negative Leukocyte Esterase and Nitrite	Total
Positive culture	23	2	25
Negative culture	225	87	312
Total	248	89	337
		**Culture 10^5^ CFU/mL**	**95% CI**
Sensitivity		92.0%	74.0–99.0%
Specificity		27.9%	23.0–33.2%
Positive likelihood ratio		1.28	1.12–1.46
Negative likelihood ratio		0.29	0.08–1.10
Positive predictive value		9.27%	8.2–10.5%
Negative predictive value		97.8%	91.9–99.4%
Total accuracy		32.6%	27.7–37.9%

**Table 4:** Performance of both the positive leukocyte esterase and positive nitrite dipstick tests in pregnant women (*N* = 337).

	Positive Leukocyte Esterase and Nitrite	Negative Leukocyte Esterase and/or Nitrite	Total
Positive culture	13	12	25
Negative culture	54	258	312
Total	67	270	337
		**Culture 10^5^ CFU/mL**	**95% CI**
Sensitivity		52.0%	31.3–72.2%
Specificity		82.7%	78.0–86.7%
Positive likelihood ratio		3.0	1.92–4.70
Negative likelihood ratio		0.58	0.38–0.88
Positive predictive value		19.4%	13.3–27.4%
Negative predictive value		95.6%	93.4–97.0%
Total accuracy		80.4%	75.8–84.5%

## Data Availability

The data presented in this study are available on request from the corresponding author.

## Supplementary Materials

The following supporting information can be downloaded at: https://www.mdpi.com/article/10.3390/antibiotics13060567/s1. Supplementary Table S1: Performance of the leukocyte esterase dipstick test in pregnant women (*N* = 718) with a cut-off of ≥10^4^ CFU/mL for the urine culture; Supplementary Table S2: Bacteria present in urine culture; Supplementary Table S3: Performance of the nitrite dipstick test in pregnant women (*N* = 337) with a cut-off of ≥10^4^ CFU/mL for the urine culture; Supplementary Table S4: Performance of the leukocyte esterase and/or nitrite dipstick tests in pregnant women (*N* = 337) with a cut-off of ≥10^4^ CFU/mL for the urine culture; Supplementary Table S5: Performance of the positive leukocyte esterase and nitrite dipstick tests in pregnant women (*N* = 337) with a cut-off of ≥10^4^ CFU/mL for the urine culture.

## Abbreviations

UTI	Urinary tract infection
CFU	Colony forming units
DOT	Diagnosis treatment combination on the way to transparency
