# Supplementary material for: Diagnostic Accuracy of Urine Dipsticks for Urinary Tract Infection Diagnosis during Pregnancy: A Retrospective Cohort Study
Source: Antibiotics (Basel). 2024 Jun 19;13(6):567. doi: 10.3390/antibiotics13060567 (PMC11200439; doi:10.3390/antibiotics13060567)
Supplement: Supplementary file 1 [file antibiotics-13-00567-s001.zip › antibiotics-3004224-supplementary.pdf]

antibiotics

MDPI

Supplementary Table S1. Performance of the leukocyte esterase dipstick test in pregnant women (N = 718) with a cut-off of ≥10^4 CFU/mL for the urine culture.

	Positive leukocyte esterase	Negative leukocyte esterase	Total
Positive culture	92	31	123
Negative culture	344	251	595
Total	436	282	718
		**Culture 10^4 CFU/mL**	**95% CI**
Sensitivity		74.8%	66.2–82.2%
Specificity		42.2%	38.2–46.3%
Positive likelihood ratio		1.29	1.14–1.46
Negative likelihood ratio		0.60	0.43–0.82
Positive predictive value		21.1%	19.1–23.2%
Negative predictive value		89.0%	85.5–91.8%
Total accuracy		47.8%	44.1–51.5%

Supplementary Table S2. Bacteria present in urine culture.

Bacteria	≥10^5 CFU/mL % N (%)	≥10^4 CFU/mL N (%)
Escherichia coli	35 (71)	73 (59)
Klebsiella pneumoniae	10 (20)	14 (11)
Streptococcus agalactiae	0 (0)	14 (11)
Enterococcus faecalis	1 (2)	9 (7)
Proteus mirabilis	0 (0)	3 (2)
Staphylococcus saprophyticus	0 (0)	2 (2)
Klebsiella oxytoca	1 (2)	1 (1)
Serratia marcescens	1 (2)	1 (1)
Streptococcus mitis	1 (2)	1 (1)
Other	0 (0)	5 (4)
Total	49	123

Supplementary Table S3. Performance of the nitrite dipstick test in pregnant women (*N* = 337) with a cut-off of ≥10^4 CFU/mL for the urine culture.

	**Positive nitrite**	**Negative nitrite**	**Total**
Positive culture	31	26	57
Negative culture	70	210	280
Total	101	236	337
		**Culture 10^4 CFU/mL**	**95% CI**
Sensitivity		54.4%	40.7–67.6%
Specificity		75.0%	69.5–80.0%
Positive likelihood ratio		2.18	1.59–2.97
Negative likelihood ratio		0.61	0.45–0.81
Positive predictive value		30.7%	24.5–37.7%
Negative predictive value		89.0%	85.8–91.5%
Total accuracy		71.5%	66.4–76.3%

Supplementary Table S4. Performance of the leukocyte esterase and/or nitrite dipstick tests in pregnant women (*N* = 337) with a cut-off of ≥10^4 CFU/mL for the urine culture.

	**Positive leukocyte esterase and/or nitrite**	**Negative leukocyte esterase and nitrite**	**Total**
Positive culture	51	6	57
Negative culture	197	83	280
Total	248	89	337
		**Culture 10^4 CFU/mL**	**95% CI**
Sensitivity		89.5%	78.5–96.0%
Specificity		29.6%	24.4–35.3%
Positive likelihood ratio		1.27	1.13–1.43
Negative likelihood ratio		0.36	0.16–0.77
Positive predictive value		20.6%	18.7–22.5%
Negative predictive value		93.2%	86.4–96.8%
Total accuracy		39.8%	34.5–45.2%

Supplementary Table S5. Performance of the positive leukocyte esterase and nitrite dipstick tests in pregnant women ($N = 337$) with a cut-off of ≥10^4 CFU/mL for the urine culture.

	**Positive leukocyte esterase and nitrite**	**Negative leukocyte esterase and/or nitrite**	**Total**
Positive culture	20	37	57
Negative culture	47	233	280
Total	67	270	337
		**Culture 10^4 CFU/mL**	**95% CI**
Sensitivity		35.1%	22.9–48.9%
Specificity		83.2%	78.3–87.4%
Positive likelihood ratio		2.09	1.35–3.24
Negative likelihood ratio		0.78	0.64–0.95
Positive predictive value		29.9%	21.5–39.8%
Negative predictive value		86.3%	83.8–88.5%
Total accuracy		75.1%	70.1–79.6%
